# Supplementary material for: Fractal Dimension, Circularity, and Solidity of Cell Clusters in Liquid-Based Endometrial Cytology Are Potentially Useful for Endometrial Cancer Detection and Prognosis Prediction
Source: Cancers (Basel). 2024 Jul 6;16(13):2469. doi: 10.3390/cancers16132469 (PMC11240598; doi:10.3390/cancers16132469)
Supplement: Supplementary file 1 [file cancers-16-02469-s001.zip › EM_Sup_table.pptx]

## Slide 1
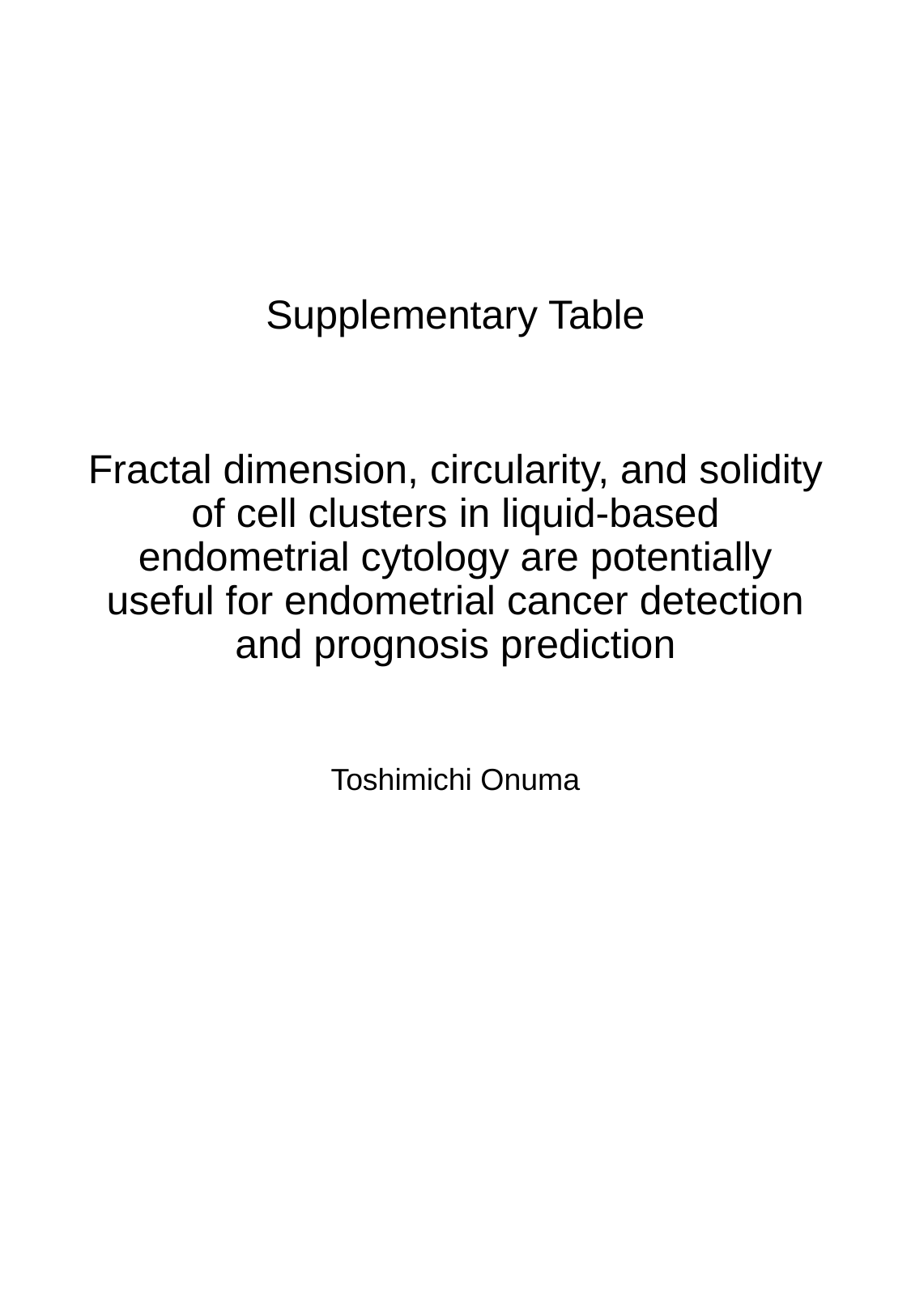

# Fractal dimension, circularity, and solidity of cell clusters in liquid-based endometrial cytology are potentially useful for endometrial cancer detection and prognosis prediction
Supplementary Table
Toshimichi Onuma

## Slide 2
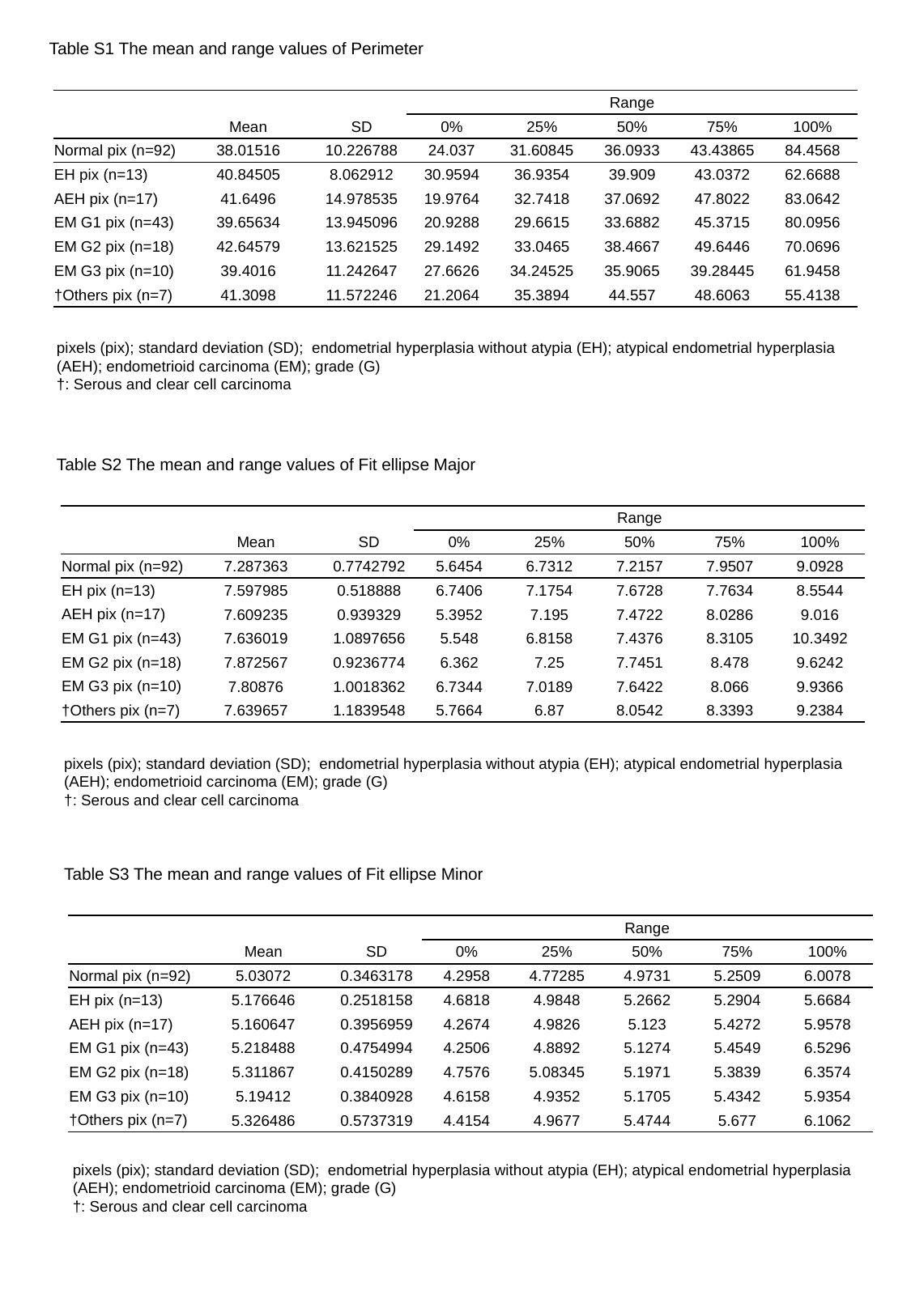

Table S1 The mean and range values of Perimeter
| | | | | | Range | | |
| --- | --- | --- | --- | --- | --- | --- | --- |
| | Mean | SD | 0% | 25% | 50% | 75% | 100% |
| Normal pix (n=92) | 38.01516 | 10.226788 | 24.037 | 31.60845 | 36.0933 | 43.43865 | 84.4568 |
| EH pix (n=13) | 40.84505 | 8.062912 | 30.9594 | 36.9354 | 39.909 | 43.0372 | 62.6688 |
| AEH pix (n=17) | 41.6496 | 14.978535 | 19.9764 | 32.7418 | 37.0692 | 47.8022 | 83.0642 |
| EM G1 pix (n=43) | 39.65634 | 13.945096 | 20.9288 | 29.6615 | 33.6882 | 45.3715 | 80.0956 |
| EM G2 pix (n=18) | 42.64579 | 13.621525 | 29.1492 | 33.0465 | 38.4667 | 49.6446 | 70.0696 |
| EM G3 pix (n=10) | 39.4016 | 11.242647 | 27.6626 | 34.24525 | 35.9065 | 39.28445 | 61.9458 |
| †Others pix (n=7) | 41.3098 | 11.572246 | 21.2064 | 35.3894 | 44.557 | 48.6063 | 55.4138 |
pixels (pix); standard deviation (SD);  endometrial hyperplasia without atypia (EH); atypical endometrial hyperplasia (AEH); endometrioid carcinoma (EM); grade (G)
†: Serous and clear cell carcinoma
Table S2 The mean and range values of Fit ellipse Major
| | | | | | Range | | |
| --- | --- | --- | --- | --- | --- | --- | --- |
| | Mean | SD | 0% | 25% | 50% | 75% | 100% |
| Normal pix (n=92) | 7.287363 | 0.7742792 | 5.6454 | 6.7312 | 7.2157 | 7.9507 | 9.0928 |
| EH pix (n=13) | 7.597985 | 0.518888 | 6.7406 | 7.1754 | 7.6728 | 7.7634 | 8.5544 |
| AEH pix (n=17) | 7.609235 | 0.939329 | 5.3952 | 7.195 | 7.4722 | 8.0286 | 9.016 |
| EM G1 pix (n=43) | 7.636019 | 1.0897656 | 5.548 | 6.8158 | 7.4376 | 8.3105 | 10.3492 |
| EM G2 pix (n=18) | 7.872567 | 0.9236774 | 6.362 | 7.25 | 7.7451 | 8.478 | 9.6242 |
| EM G3 pix (n=10) | 7.80876 | 1.0018362 | 6.7344 | 7.0189 | 7.6422 | 8.066 | 9.9366 |
| †Others pix (n=7) | 7.639657 | 1.1839548 | 5.7664 | 6.87 | 8.0542 | 8.3393 | 9.2384 |
pixels (pix); standard deviation (SD);  endometrial hyperplasia without atypia (EH); atypical endometrial hyperplasia (AEH); endometrioid carcinoma (EM); grade (G)
†: Serous and clear cell carcinoma
Table S3 The mean and range values of Fit ellipse Minor
| | | | | | Range | | |
| --- | --- | --- | --- | --- | --- | --- | --- |
| | Mean | SD | 0% | 25% | 50% | 75% | 100% |
| Normal pix (n=92) | 5.03072 | 0.3463178 | 4.2958 | 4.77285 | 4.9731 | 5.2509 | 6.0078 |
| EH pix (n=13) | 5.176646 | 0.2518158 | 4.6818 | 4.9848 | 5.2662 | 5.2904 | 5.6684 |
| AEH pix (n=17) | 5.160647 | 0.3956959 | 4.2674 | 4.9826 | 5.123 | 5.4272 | 5.9578 |
| EM G1 pix (n=43) | 5.218488 | 0.4754994 | 4.2506 | 4.8892 | 5.1274 | 5.4549 | 6.5296 |
| EM G2 pix (n=18) | 5.311867 | 0.4150289 | 4.7576 | 5.08345 | 5.1971 | 5.3839 | 6.3574 |
| EM G3 pix (n=10) | 5.19412 | 0.3840928 | 4.6158 | 4.9352 | 5.1705 | 5.4342 | 5.9354 |
| †Others pix (n=7) | 5.326486 | 0.5737319 | 4.4154 | 4.9677 | 5.4744 | 5.677 | 6.1062 |
pixels (pix); standard deviation (SD);  endometrial hyperplasia without atypia (EH); atypical endometrial hyperplasia (AEH); endometrioid carcinoma (EM); grade (G)
†: Serous and clear cell carcinoma

## Slide 3
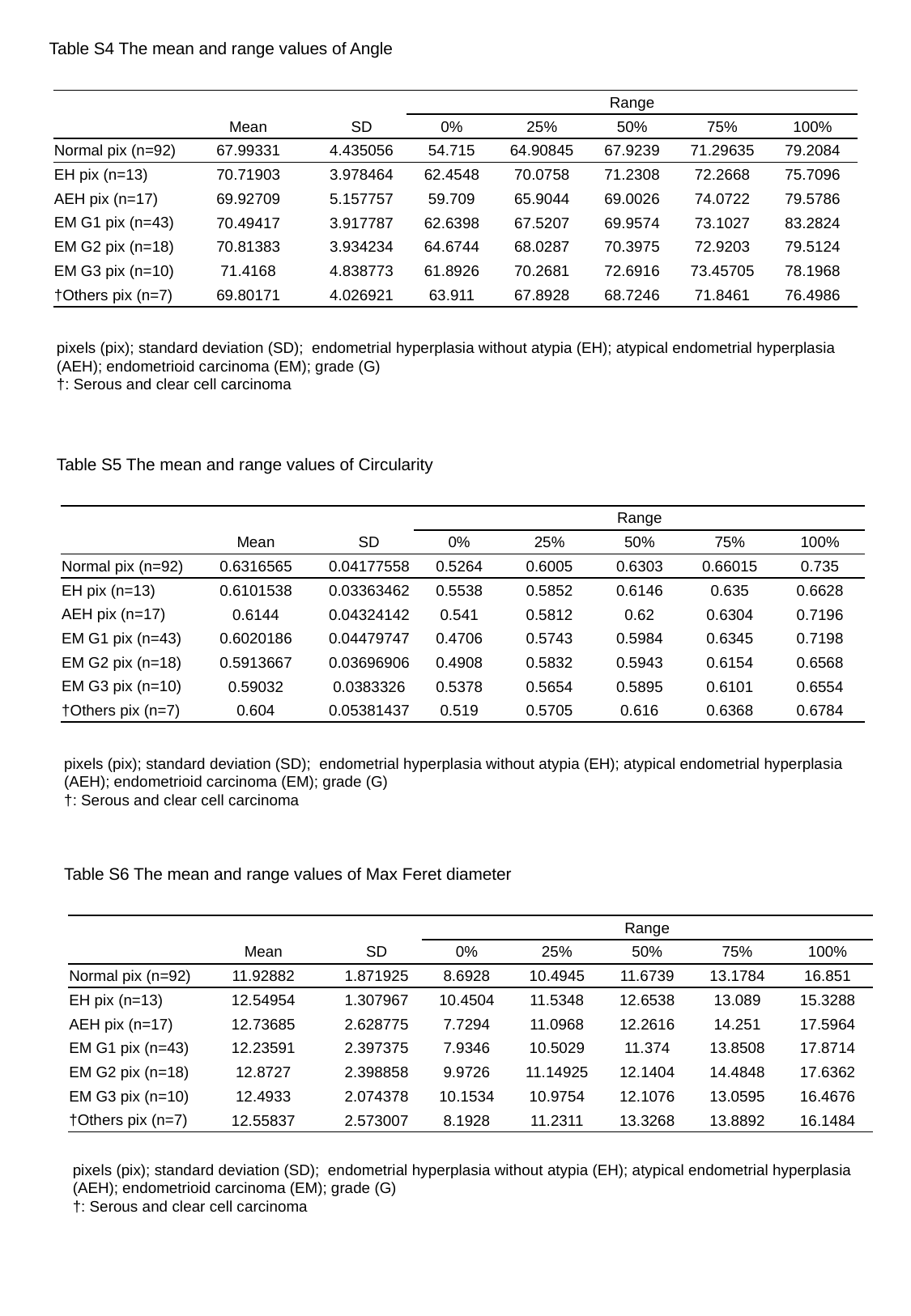

Table S4 The mean and range values of Angle
| | | | | | Range | | |
| --- | --- | --- | --- | --- | --- | --- | --- |
| | Mean | SD | 0% | 25% | 50% | 75% | 100% |
| Normal pix (n=92) | 67.99331 | 4.435056 | 54.715 | 64.90845 | 67.9239 | 71.29635 | 79.2084 |
| EH pix (n=13) | 70.71903 | 3.978464 | 62.4548 | 70.0758 | 71.2308 | 72.2668 | 75.7096 |
| AEH pix (n=17) | 69.92709 | 5.157757 | 59.709 | 65.9044 | 69.0026 | 74.0722 | 79.5786 |
| EM G1 pix (n=43) | 70.49417 | 3.917787 | 62.6398 | 67.5207 | 69.9574 | 73.1027 | 83.2824 |
| EM G2 pix (n=18) | 70.81383 | 3.934234 | 64.6744 | 68.0287 | 70.3975 | 72.9203 | 79.5124 |
| EM G3 pix (n=10) | 71.4168 | 4.838773 | 61.8926 | 70.2681 | 72.6916 | 73.45705 | 78.1968 |
| †Others pix (n=7) | 69.80171 | 4.026921 | 63.911 | 67.8928 | 68.7246 | 71.8461 | 76.4986 |
pixels (pix); standard deviation (SD);  endometrial hyperplasia without atypia (EH); atypical endometrial hyperplasia (AEH); endometrioid carcinoma (EM); grade (G)
†: Serous and clear cell carcinoma
Table S5 The mean and range values of Circularity
| | | | | | Range | | |
| --- | --- | --- | --- | --- | --- | --- | --- |
| | Mean | SD | 0% | 25% | 50% | 75% | 100% |
| Normal pix (n=92) | 0.6316565 | 0.04177558 | 0.5264 | 0.6005 | 0.6303 | 0.66015 | 0.735 |
| EH pix (n=13) | 0.6101538 | 0.03363462 | 0.5538 | 0.5852 | 0.6146 | 0.635 | 0.6628 |
| AEH pix (n=17) | 0.6144 | 0.04324142 | 0.541 | 0.5812 | 0.62 | 0.6304 | 0.7196 |
| EM G1 pix (n=43) | 0.6020186 | 0.04479747 | 0.4706 | 0.5743 | 0.5984 | 0.6345 | 0.7198 |
| EM G2 pix (n=18) | 0.5913667 | 0.03696906 | 0.4908 | 0.5832 | 0.5943 | 0.6154 | 0.6568 |
| EM G3 pix (n=10) | 0.59032 | 0.0383326 | 0.5378 | 0.5654 | 0.5895 | 0.6101 | 0.6554 |
| †Others pix (n=7) | 0.604 | 0.05381437 | 0.519 | 0.5705 | 0.616 | 0.6368 | 0.6784 |
pixels (pix); standard deviation (SD);  endometrial hyperplasia without atypia (EH); atypical endometrial hyperplasia (AEH); endometrioid carcinoma (EM); grade (G)
†: Serous and clear cell carcinoma
Table S6 The mean and range values of Max Feret diameter
| | | | | | Range | | |
| --- | --- | --- | --- | --- | --- | --- | --- |
| | Mean | SD | 0% | 25% | 50% | 75% | 100% |
| Normal pix (n=92) | 11.92882 | 1.871925 | 8.6928 | 10.4945 | 11.6739 | 13.1784 | 16.851 |
| EH pix (n=13) | 12.54954 | 1.307967 | 10.4504 | 11.5348 | 12.6538 | 13.089 | 15.3288 |
| AEH pix (n=17) | 12.73685 | 2.628775 | 7.7294 | 11.0968 | 12.2616 | 14.251 | 17.5964 |
| EM G1 pix (n=43) | 12.23591 | 2.397375 | 7.9346 | 10.5029 | 11.374 | 13.8508 | 17.8714 |
| EM G2 pix (n=18) | 12.8727 | 2.398858 | 9.9726 | 11.14925 | 12.1404 | 14.4848 | 17.6362 |
| EM G3 pix (n=10) | 12.4933 | 2.074378 | 10.1534 | 10.9754 | 12.1076 | 13.0595 | 16.4676 |
| †Others pix (n=7) | 12.55837 | 2.573007 | 8.1928 | 11.2311 | 13.3268 | 13.8892 | 16.1484 |
pixels (pix); standard deviation (SD);  endometrial hyperplasia without atypia (EH); atypical endometrial hyperplasia (AEH); endometrioid carcinoma (EM); grade (G)
†: Serous and clear cell carcinoma

## Slide 4
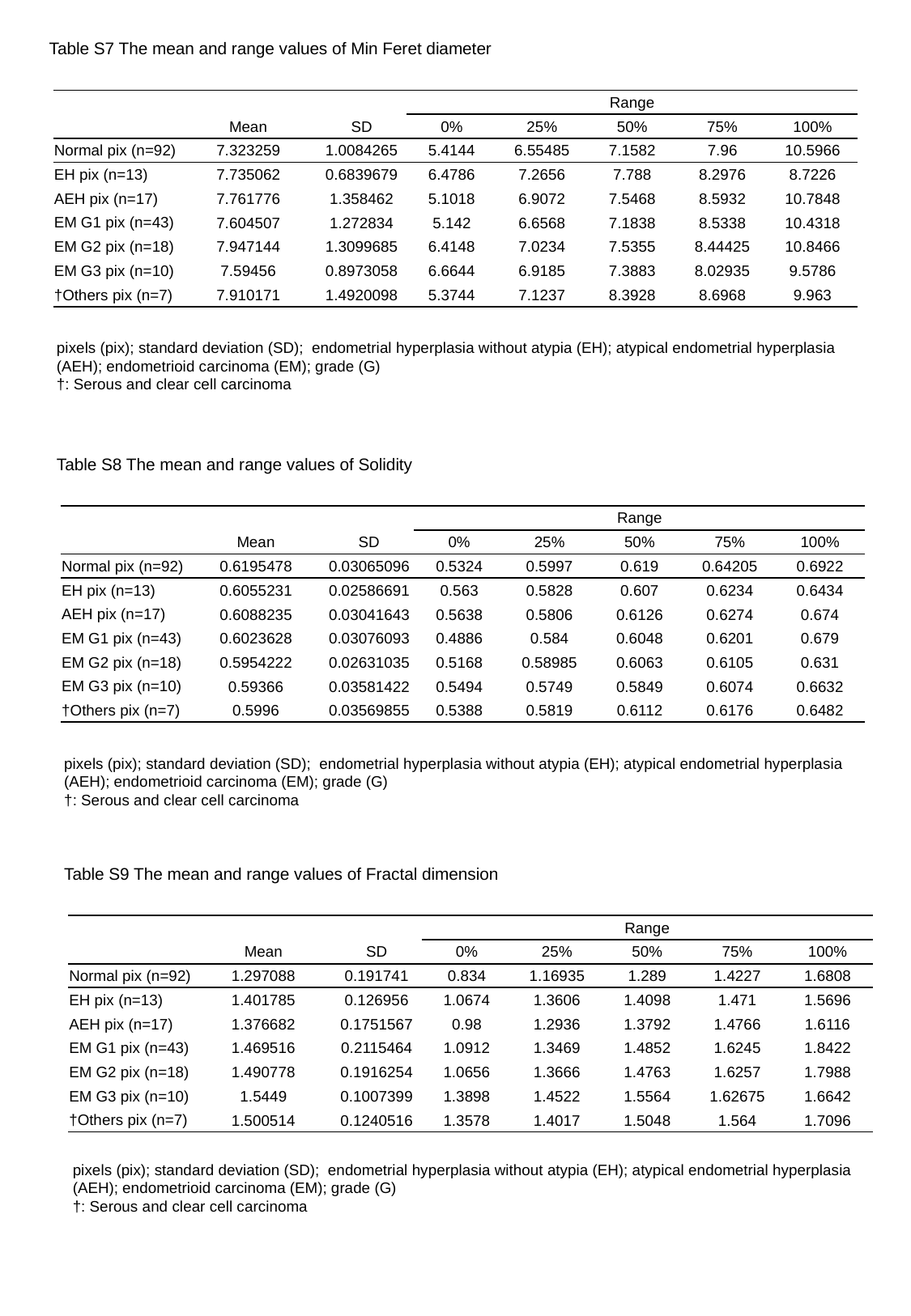

Table S7 The mean and range values of Min Feret diameter
| | | | | | Range | | |
| --- | --- | --- | --- | --- | --- | --- | --- |
| | Mean | SD | 0% | 25% | 50% | 75% | 100% |
| Normal pix (n=92) | 7.323259 | 1.0084265 | 5.4144 | 6.55485 | 7.1582 | 7.96 | 10.5966 |
| EH pix (n=13) | 7.735062 | 0.6839679 | 6.4786 | 7.2656 | 7.788 | 8.2976 | 8.7226 |
| AEH pix (n=17) | 7.761776 | 1.358462 | 5.1018 | 6.9072 | 7.5468 | 8.5932 | 10.7848 |
| EM G1 pix (n=43) | 7.604507 | 1.272834 | 5.142 | 6.6568 | 7.1838 | 8.5338 | 10.4318 |
| EM G2 pix (n=18) | 7.947144 | 1.3099685 | 6.4148 | 7.0234 | 7.5355 | 8.44425 | 10.8466 |
| EM G3 pix (n=10) | 7.59456 | 0.8973058 | 6.6644 | 6.9185 | 7.3883 | 8.02935 | 9.5786 |
| †Others pix (n=7) | 7.910171 | 1.4920098 | 5.3744 | 7.1237 | 8.3928 | 8.6968 | 9.963 |
pixels (pix); standard deviation (SD);  endometrial hyperplasia without atypia (EH); atypical endometrial hyperplasia (AEH); endometrioid carcinoma (EM); grade (G)
†: Serous and clear cell carcinoma
Table S8 The mean and range values of Solidity
| | | | | | Range | | |
| --- | --- | --- | --- | --- | --- | --- | --- |
| | Mean | SD | 0% | 25% | 50% | 75% | 100% |
| Normal pix (n=92) | 0.6195478 | 0.03065096 | 0.5324 | 0.5997 | 0.619 | 0.64205 | 0.6922 |
| EH pix (n=13) | 0.6055231 | 0.02586691 | 0.563 | 0.5828 | 0.607 | 0.6234 | 0.6434 |
| AEH pix (n=17) | 0.6088235 | 0.03041643 | 0.5638 | 0.5806 | 0.6126 | 0.6274 | 0.674 |
| EM G1 pix (n=43) | 0.6023628 | 0.03076093 | 0.4886 | 0.584 | 0.6048 | 0.6201 | 0.679 |
| EM G2 pix (n=18) | 0.5954222 | 0.02631035 | 0.5168 | 0.58985 | 0.6063 | 0.6105 | 0.631 |
| EM G3 pix (n=10) | 0.59366 | 0.03581422 | 0.5494 | 0.5749 | 0.5849 | 0.6074 | 0.6632 |
| †Others pix (n=7) | 0.5996 | 0.03569855 | 0.5388 | 0.5819 | 0.6112 | 0.6176 | 0.6482 |
pixels (pix); standard deviation (SD);  endometrial hyperplasia without atypia (EH); atypical endometrial hyperplasia (AEH); endometrioid carcinoma (EM); grade (G)
†: Serous and clear cell carcinoma
Table S9 The mean and range values of Fractal dimension
| | | | | | Range | | |
| --- | --- | --- | --- | --- | --- | --- | --- |
| | Mean | SD | 0% | 25% | 50% | 75% | 100% |
| Normal pix (n=92) | 1.297088 | 0.191741 | 0.834 | 1.16935 | 1.289 | 1.4227 | 1.6808 |
| EH pix (n=13) | 1.401785 | 0.126956 | 1.0674 | 1.3606 | 1.4098 | 1.471 | 1.5696 |
| AEH pix (n=17) | 1.376682 | 0.1751567 | 0.98 | 1.2936 | 1.3792 | 1.4766 | 1.6116 |
| EM G1 pix (n=43) | 1.469516 | 0.2115464 | 1.0912 | 1.3469 | 1.4852 | 1.6245 | 1.8422 |
| EM G2 pix (n=18) | 1.490778 | 0.1916254 | 1.0656 | 1.3666 | 1.4763 | 1.6257 | 1.7988 |
| EM G3 pix (n=10) | 1.5449 | 0.1007399 | 1.3898 | 1.4522 | 1.5564 | 1.62675 | 1.6642 |
| †Others pix (n=7) | 1.500514 | 0.1240516 | 1.3578 | 1.4017 | 1.5048 | 1.564 | 1.7096 |
pixels (pix); standard deviation (SD);  endometrial hyperplasia without atypia (EH); atypical endometrial hyperplasia (AEH); endometrioid carcinoma (EM); grade (G)
†: Serous and clear cell carcinoma
