# Supplementary material for: Fractal Dimension, Circularity, and Solidity of Cell Clusters in Liquid-Based Endometrial Cytology Are Potentially Useful for Endometrial Cancer Detection and Prognosis Prediction
Source: Cancers (Basel). 2024 Jul 6;16(13):2469. doi: 10.3390/cancers16132469 (PMC11240598; doi:10.3390/cancers16132469)
Supplement: Supplementary file 1 [file cancers-16-02469-s001.zip › Rev_Sup_Fig_EM_cytology.pptx]

## Slide 1
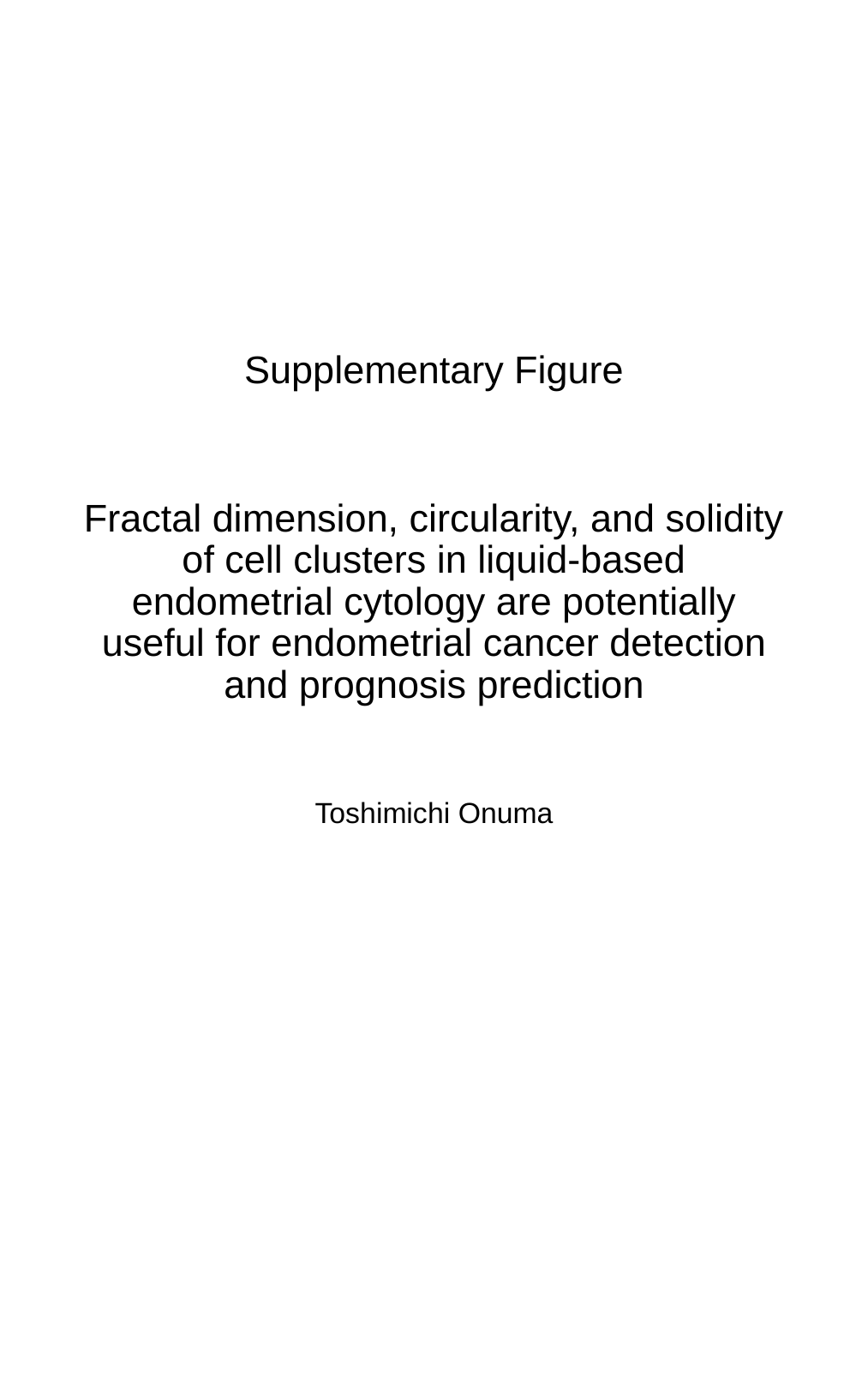

# Fractal dimension, circularity, and solidity of cell clusters in liquid-based endometrial cytology are potentially useful for endometrial cancer detection and prognosis prediction
Supplementary Figure
Toshimichi Onuma

## Slide 2
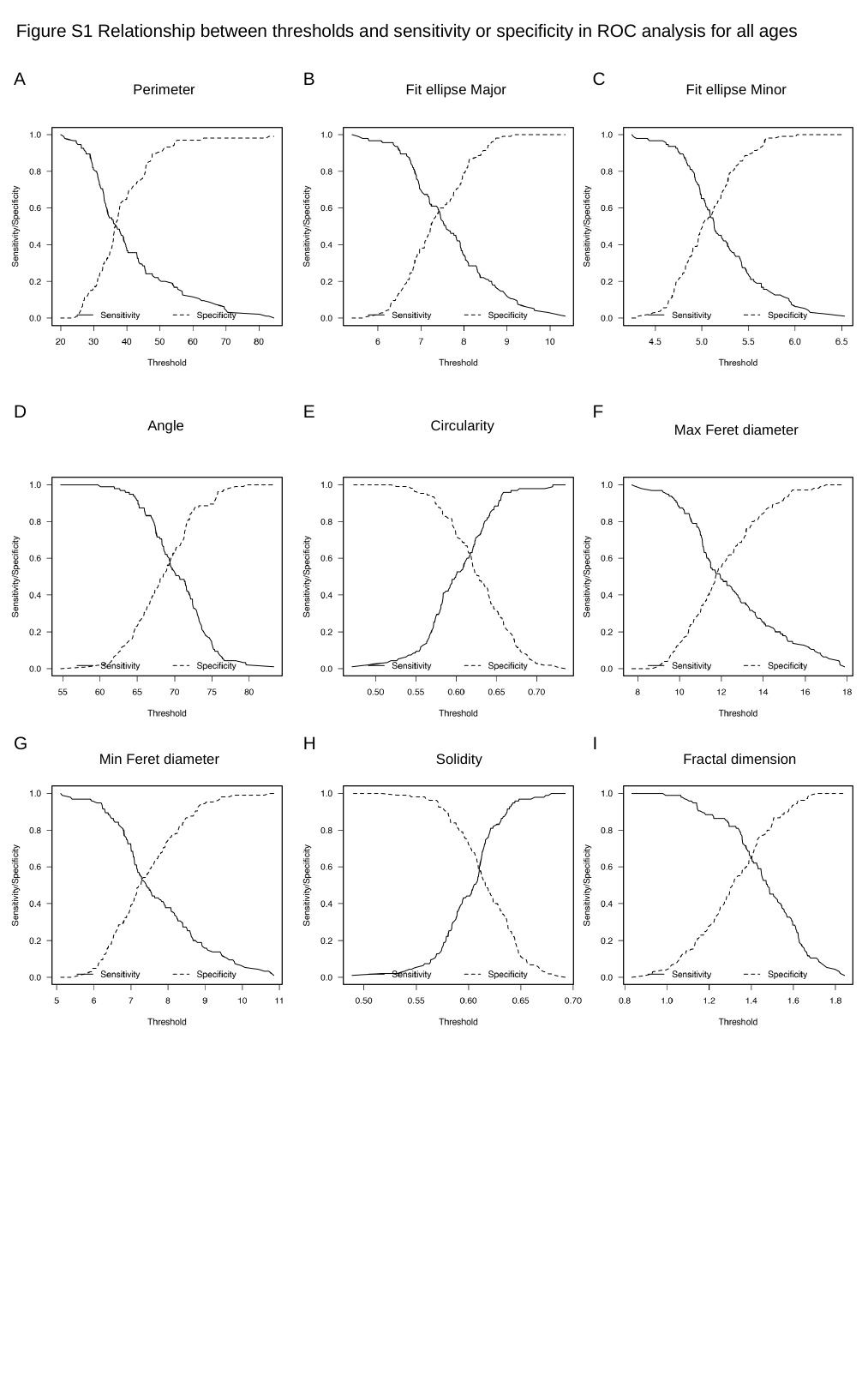

Figure S1 Relationship between thresholds and sensitivity or specificity in ROC analysis for all ages
A
B
C
Perimeter
Fit ellipse Major
Fit ellipse Minor
D
E
F
Angle
Circularity
Max Feret diameter
G
H
I
Min Feret diameter
Solidity
Fractal dimension

## Slide 3
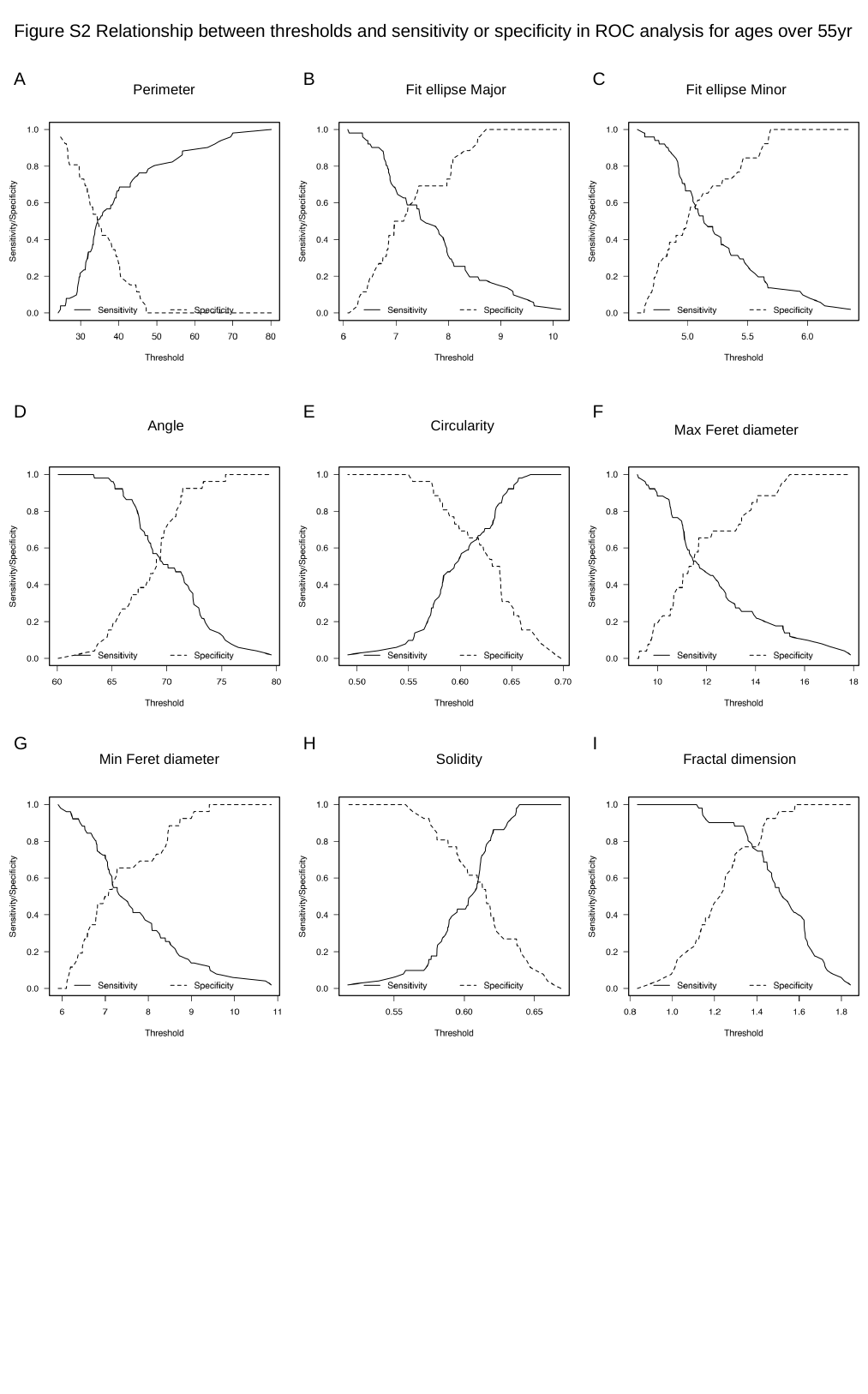

Figure S2 Relationship between thresholds and sensitivity or specificity in ROC analysis for ages over 55yr
A
B
C
Perimeter
Fit ellipse Major
Fit ellipse Minor
D
E
F
Angle
Circularity
Max Feret diameter
G
H
I
Min Feret diameter
Solidity
Fractal dimension
